# Aging Affects Insulin Resistance, Insulin Secretion, and Glucose Effectiveness in Subjects with Normal Blood Glucose and Body Weight

**DOI:** 10.3390/diagnostics13132158

**Published:** 2023-06-24

**Authors:** Li-Ying Huang, Chi-Hao Liu, Fang-Yu Chen, Chun-Heng Kuo, Pietro Pitrone, Jhih-Syuan Liu

**Affiliations:** 1 Division of Endocrinology and Metabolism, Department of Internal Medicine, Fu Jen Catholic University Hospital, New Taipei 24352, Taiwan; liyinghuang@yahoo.com (L.-Y.H.); julia0770@yahoo.com.tw (F.-Y.C.); cpp0103@gmail.com (C.-H.K.); 2 Division of Nephrology, Department of Medicine, Kaohsiung Armed Forces General Hospital, Kaohsiung 80284, Taiwan; colinliu1201@gmail.com; 3 Department of Biomedical and Dental Sciences and Morpho-Functional Imaging, University of Messina, 98158 Messina, Italy; pieropitrone@live.it; 4 Division of Endocrinology and Metabolism, Department of Internal Medicine, Tri-Service General Hospital, National Defense Medical Center, Taipei 11490, Taiwan

**Keywords:** first-phase insulin secretion, second-phase insulin secretion, insulin resistance, glucose effectiveness, type 2 diabetes

## Abstract

Aim: Several studies have demonstrated that factors including diabetes, including insulin resistance (IR), glucose effectiveness (GE), and the first and second phase of insulin secretion (FPIS, SPIS) could easily be calculated using basic characteristics and biochemistry profiles. Aging is accompanied by deteriorations of insulin resistance (IR) and insulin secretion. However, little is known about the roles of aging in the different phases of insulin secretion (ISEC), i.e., the first and second phase of insulin secretion (FPIS, SPIS), and glucose effectiveness (GE). Methods: In total, 169 individuals (43 men and 126 women) recruited from the data bank of the Meei-Jaw (MJ) Health Screening Center and Cardinal Tien Hospital Data Access Center between 1999 and 2008, with a similar fasting plasma glucose (FPG: 90 mg/dL) and BMI (men: 23 kg/m^2^, women 22 kg/m^2^) were enrolled. The IR, FPIS, SPIS, and GE were estimated using our previously developed equations shown below. Pearson correlation analysis was conducted to assess the correlations between age and four diabetes factors (DFs: IR, FPIS, SPIS, and GE). The equations that are used to calculate the DF in the present study were built and published by our group. Results: The age of the participants ranged from 18 to 78 years. Men had higher FPIS but lower HDL-C levels than women (2.067 ± 0.159, 1.950 ± 0.186 μU/min and 1.130 ± 0.306, 1.348 ± 0.357 mmol/dl, accordingly). The results of the Pearson correlation revealed that age was negatively related to the IR and GE in both genders (IR: r = −0.39, *p* < 0.001 for men, r = −0.24, *p* < 0.003 for women; GE: r = 0.66, *p* < 0.001 for men, r = 0.78, *p* < 0.001 for women). At the same time, the FPIS was also only found to be negatively correlated with age in females (r = −0.238, *p* = 0.003), but there was no difference in the SPIS and age among both genders. Conclusions: We have found that in Chinese subjects with a normal FPG level (90 mg/dL) and body mass index (men: 23 kg/m^2^, women: 22: kg/m^2^), age is negatively related to the IR and GE among both genders. Only the FPIS was found to be negatively related to age in women. The tightness of their relationships, from the highest to the lowest, are GE, FPIS, and IR. These results should be interpreted with caution because of the small sample size.

## 1. Introduction

The incidence of type 2 diabetes (T2D) has been increasing recently, both in developed and developing countries. Using the World Health Organization diagnostic criteria, the estimated prevalence in the whole world was 10.08% for men and 9.14% for women [1]. In Taiwan, a similar trend could also be noted; it has increased from 5.1% to 12.8% from 1970 to 1993. This could be contributed by two factors: first, the aging of our society. Because of the decreasing birth rate, Taiwan has become an aging society since March 2018. A seventh of the whole population is over 65 years old. Evidence has shown that Taiwan will become an aging society within 8 years [2]. Second, the Westernization of Taiwanese lifestyle causes obesity in the country. Chang et al. reported that although the prevalence of overweight was relatively stable in recent years, obesity (BMI ≥ 25 kg/m^2^) and morbid obesity (BMI ≥ 35 kg/m^2^) increased sharply from 1993 to 2014 (11.8% to 22.0%, and 0.4% to 1.4%, respectively). Morbidly obesity subjects had a lower educational level, income, and physical activity. Both obese and morbidly obese subjects tended to over-consume red meat, processed products, and sweetened beverage [3].

It is worrisome that T2D could cause a serious burden not only to individuals, but also to their families, as well as the health provider. At present, it is the fifth top cause of death in Taiwan, according to the statistics of the National Health Institute of Taiwan [4]. Therefore, the aim of this study is to further understand the underlying pathophysiology of T2D before it becomes an urgent issue for the government of each country.

It is generally agreed that the natural course of diabetes starts with increased insulin resistance (IR). Insulin secretion (ISEC) rises to compensate for this perturbation. However, decades of this ongoing compensation eventually results in decompensation. This is when overt clinical diabetes can be diagnosed [5,6].

Physiologically, there is a rapid burst within the first ten minutes of β-cells secreting insulin, regarded as the first-phase insulin secretion (FPIS). After this peak, there is a second slow-rising plateau, i.e., second-phase insulin secretion (SPIS), which lasts until plasma glucose returns to its baseline [7]. This second part of insulin is newly produced insulin by β-cells. At present, most studies only focus on the roles of FPIS. This clinical observation indicates that years after the initiation of diabetes, glucose homeostasis should be maintained by the SPIS. Surprisingly, little is known about the influences of SPIS, even though it could have an important role in glucose homeostasis [8].

Alongside aforementioned insulin-related factors, non-insulin-dependent glucose uptake can impact plasma glucose levels. This pathway of glucose metabolism is known as glucose effectiveness (GE) and can be considered as glucose’s ability to enhance its cellular uptake and suppress endogenous hepatic glucose output under basal insulin levels [9,10]. Similar to the SPIS, the role of GE is also underestimated. It can be easily understood from the clinical observation that in long-term diabetes patients, both the IR and ISEC deteriorate to a very severe condition; the only left factor which could decrease the plasma glucose level is GE. Some researchers also consider the GE crucial for maintaining the glucose level within a normal range in these subjects. However, only limited numbers of studies focused on the GE [11,12,13]. In the present study, we named these four factors (IR, FPIS, SPIS, and GE) ‘diabetes factors’ (DFs).

Aging has certain effects on glucose metabolism. For instance, the mean age of the onset of T2D in Taiwan is approximately 58 years [14]. Simultaneously, the incidence of diabetes is positively related to an increase in age [15], which can be explained by the fact that ISEC decreases with age, leading to decompensation against the IR, as aforementioned [16]. Ropelle et al. showed that aging in mice was associated with increased inducible nitric oxide synthase (iNOS) expression and insulin receptor S-nitrosylation, which eventually increased the IR [17]. Many studies have explored the ISEC relationship with aging. Even though the effects of age on DFs are well-appreciated; however, the extents and relative weights of their relationships remain unknown. Evidence has shown that DFs are affected by at least three important factors: age, ambient plasma glucose, and body mass index (BMI) [18,19,20,21].

Obviously, if one wanted to explore the ‘net’ relationship between age and DFs, ambient plasma glucose and BMI would become confounding factors. In the past, most related studies used statistical methods to perform the adjustment and solve this problem. To our knowledge, no study has ever used a group of subjects with the same BMIs and ambient plasma glucose levels. Since the DFs are profoundly affected by the ambient plasma glucose level and body weight, it would be inappropriate if these confounding factors are not adjusted for.

The present study enrolled subjects with the same fasting plasma glucose (FPG) levels (90 mg/dL) and BMIs (23 kg/m^2^ in males and 22 kg/m^2^ in females). The IR, FPIS, SPIS, and GE were examined in the same individuals. Thus, the effects of aging on the four DFs could be evaluated and compared simultaneously in one subject.

## 2. Materials and Methods

### 2.1. Ethics

The subjects were recruited from the data bank of the Meei-Jaw (MJ) Health Screening Center and Cardinal Tien Hospital Data Access Center (private and primary care institution) between 1999 and 2008. The participants in this study were recruited from the data of Cardinal Tien Hospital. This is a medium-sized teaching hospital in New Taipei City, Taiwan. All participants are anonymous and informed consent was obtained before conducting the study. All participant data have been delinked to protect personal data for disclosure to other unrelated parties. Research proposals were reviewed by the institutional review board of Cardinal Tien hospital. The data did not include potentially identifying or sensitive patient information, and were not provided to a third-party organization. The study program was reviewed and approved by the Institutional Review Board of the MJ Health Screening Center of the Cardinal Tien Hospital before the study was performed. The contact information for the Cardinal Tien Hospital Data Access Committee is +886-22219331.

### 2.2. Participants

We enlisted 169 individuals (43 men and 126 women) with normal FPG levels (5.56 mmol/L) and BMIs (23 kg/m^2^ in males and 22 kg/m^2^ in females). Their ages ranged from 18 to 78 years. The subjects had no other clinical diseases, no history of diabetes mellitus or diabetic ketoacidosis, and were not using any medications known to affect insulin sensitivity (InS) or β-cell function during the study period. The BMIs were identified as body weight (kg)/height (m^2^). Waist circumferences (WCs) were measured carefully at the natural waist, which was defined as the level of the hollow molding of a laterally concave trunk. The systolic blood pressure (SBP) and diastolic blood pressure (DBP) were surveyed in the right arm of seated individuals, using a standard mercury sphygmomanometer. Blood specimens were collected from the antecubital vein for biochemical analysis.

### 2.3. Calculating the IR, FPIS, SPIS, and GE

The equations for calculating the IR, FPIS, SPIS, and GE are as follows. International units were used and a brief report is presented here to demonstrate the reliability of the equations. When conducting these studies, nearly 70% of the sample participants were used to construct the equation, and the remaining 30% were used for external validation. Therefore, the accuracies of the equations are reliable. All these four equations were published in peer-reviewed journals.

1. IR: 327 subjects were enrolled in the study. The IR was estimated using an insulin suppression test. The *r*-value between the obtained and calculated GE was 0.581 (*p* < 0.001). This can be observed in the 2013 *Journal of Diabetes Investigation*.

IR = log (1.439 + 0.018 × sex − 0.003 × age + 0.029 × BMI − 0.001 × SBP + 0.006 × DBP + 0.049 × TG − 0.046 × HDLC − 0.0116 × FPG) × 10^3.333^ [22]

2 FPIS: 186 subjects were enrolled. The FPIS was measured using an intravenous glucose tolerance test via frequent sampling. The *r*-value between the measured and calculated GE was 0.671 (*p* < 0.000). The following equation was published in the *International Journal of Endocrinology* in 2015.

FPIS = 10^(1.477−0.119×FPG+0.079×BMI−0.523×HDLC)^ [23]

3 SPIS: 82 participants were enrolled. The SPIS was measured using a modified glucose infusion test at a low dose. The *r*-value between the measured and calculated GE was 0.65 (*p* = 0.002). It was referred to in *Metabolic Syndrome and Related Disorders* in 2016.

SPIS = 10^(−2.4−0.088×FPG+0.072×BMI)^ [24]

4 GE: 227 participants were enrolled. The GE was measured using a constantly sampled intravenous glucose tolerance test. The *r*-value between the measured and calculated GE was 0.43 (*p* = 0.001). It was published in *Metabolic Syndrome and Related Disorders* in 2016.

GE = (29.196 − 0.103 × age − 2.722 × TG − 0.592 × FPG) × 10^−3^ [25]

### 2.4. Laboratory Evaluation

After ten hours of overnight fasting, blood specimens were drawn from each participant for further analysis. Plasma was centrifuged from whole blood within 1 h, and stored at −70 °C. The FPG and plasma lipid profiles were also measured. The glucose oxidase method (YSI 203 glucose analyzer; Scientific Division, Yellow Springs Instruments, Yellow Springs, OH, USA) was used to identify the FPG levels. The dry, multilayer analytical slide method, with a Fuji Dri-Chem 3000 analyzer (Fuji Photo Film, Minato-Ku, Tokyo, Japan), was used to measure the total cholesterol and triglyceride (TG) levels. An enzymatic cholesterol assay following dextran sulfate precipitation was used to define the serum high-density lipoprotein cholesterol (HDL-C) and low-density lipoprotein cholesterol (LDL-C) levels.

### 2.5. Statistical Analysis

Due to evidence showing that glucose metabolism is affected according to the gender, we analyzed our data in men and women separately.

The results of our data in this study are presented as the mean ± standard deviation. All data were tested for normal distribution with the Kolmogorov–Smirnov test, and for homogeneity of variances with Levene’s test. Student’s t-test for continuous data differences between males and females was applied. Since both age and DF are continuous variables, the relationship between them was assessed employing Pearson’s correlation. Again, this was carried out separately for men and women. The tightness of the relationship between two variables is indicated by r, and the higher the value of r, the more consistent the two variables are. All statistical tests were two-sided and *p* < 0.05 was the cutoff for statistical significance. All such statistical analyses were performed using SPSS 16.0 for Windows (SPSS, Chicago, IL, USA).

## 3. Results

This study enrolled 43 men and 126 women. Table 1 shows the demographic characteristics, biochemical data, and DFs of the participants. Notably, men had higher FPIS but lower HDL-C levels than women (2.067 ± 0.159 μU/min vs. 1.950 ± 0.186 μU/min, and 1.130 ± 0.306 mmol/L vs. 1.348 ± 0.357 mmol/L, respectively).

Table 2 presents the correlations between age and DFs. Both the IR and GE were negatively correlated with age in men. The *r*-value of GE was higher than that of IR. Similar findings were observed in women. However, the FPIS was also negatively correlated with age. The order of the *r*-values was, from highest to lowest, GE, IR, and FPIS.

**Figure 1:**
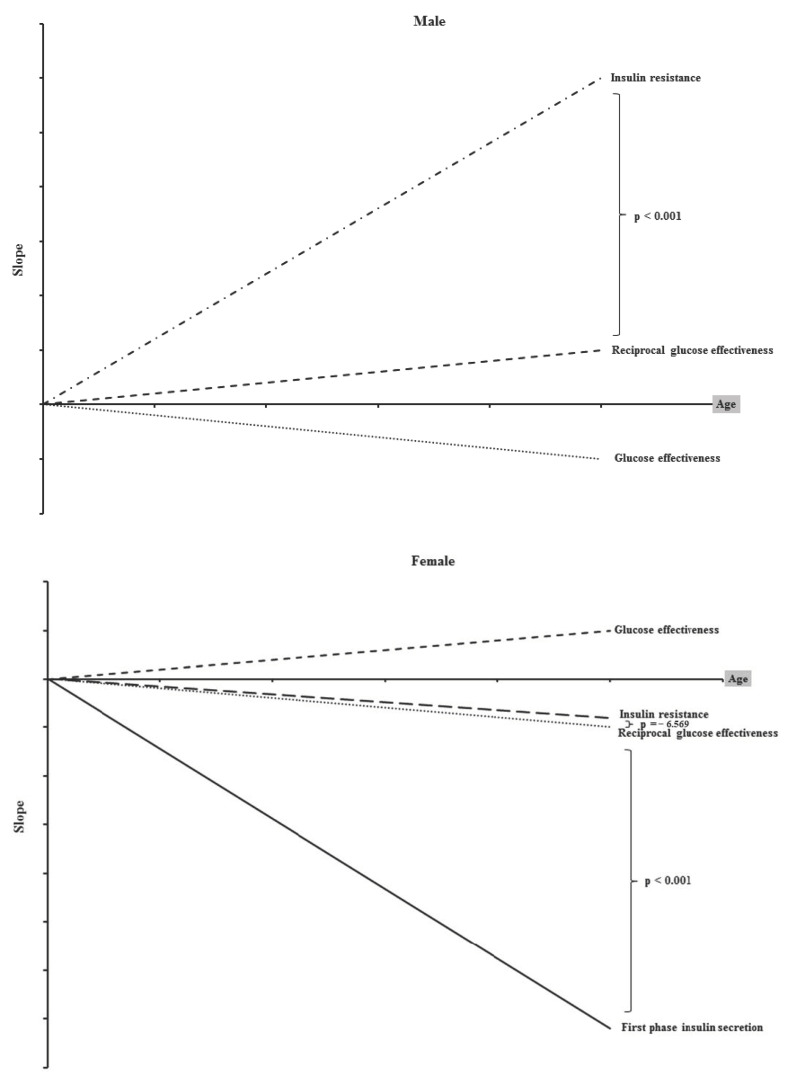
The relationships of insulin resistance, glucose effectiveness, and first-phase insulin secretion in men and women with age.

Figure 1 is the graphical demonstration of our data. The relationships (slopes) between age and IR, GE, and FPIS (only in women) are depicted. It can be noted that both the IR and GE decrease mildly as age increases. At the same time, a more drastic the decrease in the FPIS could only be noted in women. All these relationships are statistically significant.

All lines are changes in slopes of the insulin resistance (dot line), glucose effectiveness (dash line), and first-phase insulin secretions (solid line) when age increases. They are all statistically significant related to age.

## 4. Discussion

In this study, we found that the GE and IR were negatively associated with age for both sexes. A similar correlation between the FPIS and age was noted in females only. To our knowledge, this is the first study to explore the associations between age and DFs simultaneously in subjects with normal FPG levels and BMIs.

As aforementioned, it should be noted that all DFs are significantly affected by the FPG, BMI, and age. If the ‘net’ relationships between age and DFs are to be explored, the confounding influences of the BMI and FPG must be adjusted. The previously limited numbers of studies all used statistical methods to achieve the adjustment. In the present study, we enrolled subjects with exactly the same BMI and FPG, so that their complexed relationships could be clearly explored. First, it should be clarified how the FPG is affected by DFs. This issue has been discussed extensively in other studies. For instance, it is well known that the FPG is the result of the interaction between the IR and ISEC. A higher IR and lower ISEC produce a higher FPG [26]. At the same time, a higher BMI is found to be related to a higher IR [27], but its effects on the ISEC depend on whether glucose intolerance ensues. In their review, Matuszek et al. depicted that the ‘IR is a disorder secondary to obesity’. They demonstrated that the direct impact of obesity on the IR is the overload of adipose tissue and lipids, which causes an impaired glucose tolerance. Adiposity also has indirect effects via inflammation. It is a source of pro-inflammatory cytokines, impaired adipokine secretion, and it could increase the release of free fatty acids, which are well known as low-grade systemic inflammatory factors [28]. As aforementioned, the impact of obesity on the ISEC depends on the stage of glucose intolerance. In non-diabetic subjects, the higher the BMI, the higher the ISEC [29]. However, once glucose intolerance begins, the ISEC will decrease [30]. Thus, in order to obtain the pure relationships between age and DFs, we enrolled subjects with the same BMI and FPG to eliminate these confounding factors.

The notion of aging regarding the IR, ISEC, and GE has been extensively discussed previously. One of the most important studies, published in *Diabetes* in 2006, was conducted by Basu et al. By using [6-(3)H]glucose and [6,6-(2)H(2)]glucose, they observed a higher IR and lower ISEC in an older group of men, but not women. Simultaneously, the GE did not differ between older and younger subjects across both sexes. However, they only showed the differences in older and younger subjects, which is less accurate than our study [31]. The most recent article by Bacos et al. further explained the mechanisms of decreased pancreas function by demonstrating age-related DNA methylation changes in human β-cells in T2D [32]. In the past two decades, this topic has not been discussed often. Therefore, we could only find a few references. However, we believe that this does not negate the pathophysiological effects of age.

In our study, the results are presented for men and women separately. This is mainly due to the fact that gender has many influences on glucose metabolism. Mauvais-Jarvis reported that there is a significantly different response to the oral glucose tolerance test between men and women. The FPG was lower, but two hours after the challenge of glucose, the plasma glucose level was higher in women than in men [33]. There are many hypotheses attempting to explain this phenomenon. For example, less muscle mass in women, which is responsible for glucose uptake, might contribute to this diversity [34]. Moreover, gonadal hormones might play a role. In menopausal women, the FPG will decrease and, at the same time, an impaired glucose tolerance test is noted [35,36]. The aforementioned facts are the rationale for us to analyze data in men and women separately.

In the present study, age was one of the most important factors which related to DFs. However, it should be noted that, other than the chronological age evaluated here, biological differences also play important roles in affecting these factors. The biological age is estimated by biomarkers of aging and could also serve as a predictor for age-related disorders [37]. Although there was no specific study carried out to evaluate the roles of biological age on DFs, its roles could be speculated via metabolic syndrome. A vast amount of studies were carried out in this area [38,39,40]. One of the studies, with the largest cohort, produced in Korea could explicitly demonstrate the importance of biological study. Bae et al. enrolled 16,518,532 subjects. The goal was trying to develop a new index for metabolic syndrome, taking biological age into consideration. Their data showed that smoking and alcohol consumption are increased, and, at the same time, physical activity is decreased. Thus, they concluded that biological age can be a supplementary tool for evaluating and managing metabolic syndrome [41]. Even though the roles of biological age are well-established, in the present study, its effects were adjusted according to the chronological age, and other related demographic and biochemistry data.

It is generally agreed that both the IR and decreased ISEC are the most important underlying pathophysiological aspects for T2D. The simplest way to describe IR is the ‘blunting of insulin’s hypoglycemic effect’. To enter the cells, glucose needs insulin as a ‘key to open the glucose channels, which exist on the cell membrane of muscle, liver, and fat tissues [42]’. Interestingly, it should be noted that IR could trigger cardiovascular diseases via the influence of dyslipidemia, protein metabolism, oxidation, and endothelium dysfunction [43,44]. Therefore, IR is also regarded as the ‘core’ of metabolic syndrome. Other than this, some studies showed that IR is inherited from parents [45]. Evidence has shown that there is an aggregation of IR, dyslipidemia, hypertension, and hyperglycemia, which are all risk factors for cardiovascular diseases. Thus, to further understand the impacts of IR is of clinical importance.

The role of aging in IR is obvious from the clinical observation. It is well known that the average age of having diabetes is in the mid-fifties [46]. As the age increases, the prevalence of T2D and impaired glucose tolerance also becomes higher [47,48]. This phenomenon is not caused by one simple derangement, but by many factors such as increased body fat, decreased physical activity, and ISEC deficiency with aging [49]. There are still unknown areas for age-related glucose intolerance. Even so, it is surprising that there are very few studies focused on this issue. One Spanish study performed by Gayoso-Diz et al. gave some information [50]. In their study, the focus was trying to re-evaluate the cut-off point of homeostasis assessment for insulin resistance (HOMA-IR) as age increases in men and women. Their data showed that the non-linear increase in HOMA-IR could indirectly be noted in women but not in men. In another comprehensive review, Chang et al. have concluded that, from previous studies, IR decreases with age [51]. In this review, the focus was on the changes of ISEC as the age increases. Although there is no direct analysis of the relationship between age and IR, this article hints that IR decreases with age. On the other hand, in the study performed by Ferrannini et al., it was shown that IR decreased slightly with age, using the euglycemic clamp test [52]. This study was conducted in 1146 men and women, and their age ranged from 18 to 85 years. It was found that the insulin action decreased mildly with age at a rate of 0.9 mmol/min/kg every 10 years. However, after adjusting for BMI, this significance disappeared. A similar finding was also noted in Japan. By using the homeostasis model assessment and oral glucose tolerance test, Akehi et al. found the same non-significant relationship between young and old participants [16]. Interestingly, rather than using a simple correlation, they divided the study participants into six age groups and compared their results for differences in insulin concentration over glucose concentration (deltaI30/deltaG30), and the homeostasis assessment of IR. They have shown that age does not have an impact on IR. However, it is well known that both methods are surrogates for IR and less accurate compared to the euglycemic clamp study. Their findings are less persuasive. Other than these two studies, no other human study could be found in literature. In an animal model, Ropelle et al. performed a study to prove that IR is positively related to age [17]. In mice, aging is related to iNOS expression and S-nitrosation of the insulin receptor IRS-1 and AKT/PKB in skeletal muscle, along with IR. Thus, from the above evidence, we can conclude that there might be a negative relationship between IR and age. However, this correlation is a relatively weak one. A longitudinal study is needed to solve this dilemma.

It is interesting to note that there are many underlying causes of IR. The acquired causes are: excess dysfunctional adipose tissue, aging, physical inactivity, etc. At the same time, there are also genetic causes such as myotonic dystrophy, ataxia–telangiectasia, Alstom syndrome, and the Rabson–Mendenhall syndrome, etc. [53]. It is interesting to note that even Helicobacter pylori is linked to IR [54]. By using homeostasis assessment, it was shown that subjects with Helicobacter pyloric contamination had higher IR. Although we did not collect information related to Helicobacter pylori, the fact that IR is also affected by other factors should be noted.

The notion of whether the ISEC decreases with age is important and has attracted the attention of many researchers. At present, there is a consensus regarding the negative association between age and ISEC. Interestingly, some sporadic studies have shown that the response of insulin to glucose challenges is normal or even increases in elderly subjects, which is contrary to mainstay acknowledgments. However, after adjusting for confounding factors (such as adiposity), the association becomes negative and significant.

To measure the ISEC, there are several mainstay methods. The most used and also simplest one is the hemostasis assessment of insulin secretion (HOMA-IS) [55]. Although this method is easily calculated from the FPG and fasting plasma insulin, it has its drawbacks. It only indicates a static ISEC in the fasting status. This method cannot provide the whole picture of the ISEC, i.e., the dynamic changes after challenges with nutrients such as glucose and amino acid. This response to nutrient challenges is much more important in the pathophysiology of glucose hemostasis than the static status provided by HOMA-IS. The second method commonly used is an oral glucose tolerance test (OGTT). It could correct this drawback by providing information such as the ratio of plasma insulin and glucose concentration after 30 min. Of note, it reflects the ability of the ISEC response to the glucose challenge, which is a better indicator for the ISEC [56]. However, the OGTT is still limited by multiple gastrointestinal factors which could change the absorption of glucose. The other two methods that could measure the ISEC more accurately and provide both the FPIS and SPIS are the intravenous glucose tolerance test (IVGTT) and hyperglycemic clamp [57,58]. In the IVGTT, the bolus of glucose water in the vein could stimulate ISEC. The area under the plasma insulin curve before 20 min is considered the FPIS, and the plasma insulin level after this time point is the SPIS. However, it still has a defect: the most widely used protocol of IVGTT, an injection of insulin will be given, which makes the SPIS difficult to calculate. The final method and the gold standard is the hyperglycemic clamp. In this method, a bolus of glucose is given at time point 0. After this bolus, an adjustable glucose infusion is given continuously to keep the plasma glucose concentration above the original FPG level (thus, it is called hyperglycemic). Again, the first peak of the plasma insulin level is the FPIS and the afterward peak is the SPIS. Since exogenous glucose is administered throughout the whole course of the test, it is considered the most accurate method to measure the FPIS and SPIS. However, the last two methods are both labor-intensive and expensive. Thus, they could only be used in small-scale studies. In the present study, our equations for measuring the ISEC were achieved via the IVGTT. Thus, our results are more reliable than those from the HOMA-IS or OGTT.

Even though the importance of both phases of the ISEC is stressed in the previous paragraph, until now, only a handful studies have tried to investigate these two phases separately [7,59,60]. These studies were not only published in the past, but also had very small number of participants. For instance, Palmer et al. also demonstrated that the FPIS is attenuated in aged subjects. In their study, 20 g glucose water was injected and the measurement of the area under the insulin curve from 0 to 10 min was regarded as the surrogate of the FPIS. Again, only 11 young and old subjects were enrolled in that study [61]. From these previous reports, the consensus seems to be the same. Aging could decrease the ISEC. Moreover, as mentioned in the introduction, a lack of standardization of the confounding variables, such as obesity, further aggravates this dilemma. In the present study, our data showed that age is negatively related to the FPIS in women only. Thus, it could be interpreted that even though these relationships exist, their association should be weak. To explain this observation, we hypothesize that originally, the decrease in the ISEC should be more evident than we observed. However, due to IR decreases simultaneously with age, which triggers the compensation of ISEC, this masks the pure effect derived from age. In other words, the compensation attenuates the influences of age on the ISEC. Finally, although it could be argued that our equations are only surrogates for DFs, the strength of the present study is the relatively large number of participants and stringent inclusion criteria. Thus, our findings could still shed light on this area.

The importance of GE has long been underestimated. Some researchers suggested that it is responsible for nearly 99% of the glucose metabolism in T2D. Among these four DFs, GE is the least investigated one. However, its importance could be stressed through clinical observation. In many T2D patients, decades after diabetes is diagnosed, glucose could still be simply controlled by either a low dose of metformin and/or sulfonylurea. In these patients, the ISEC is already decreased to a very low level [62]. For example, from the United Kingdom Prospective Diabetes Study, the FPIS dramatically decreased even in pre-diabetes [63]. At the same time, from the aforementioned evidence, decreased in the InS in aged diabetes is also evident. Thus, the only remaining factor to maintain stable glucose homeostasis is GE. Unfortunately, little is known about the role of GE in the diverse diabetes condition.

Similar to the ISEC, the main obstacle is that the technique to measure GE is difficult [64]. At present, the most adopted method is the IVGTT. However, other than the aforementioned drawbacks, a computer program ‘minimal model’ is needed for the calculation of the InS, FPIS, and GE. According to Basu et al., in healthy subjects, GE accounts for 66% of glucose metabolism. On the other hand, in subjects with diabetes, both the InS and ISEC are all decreased to a serious level. Thus, GE begins to takeover 99% of glucose utilization. Evidence have shown that GE could be affected by the BMI level [65]. For example, Lopez suggested that there is a negative correlation between the BMI and GE [21]. However, on the contrary, Healy et al.’s data supported the opposite conclusion [66].

In the present study, our data further confirmed this observation. Other than that, it is interesting to note that the GE is most tightly related to age compared to the other three factors. Thus, the importance of GE proposed by Best et al. is further proven. It should be mentioned that one of the unique aspects of our study is that we measured the four DFs simultaneously in one person. This has never been done in other similar studies before. Thus, we believe that our findings are novice and worthy of further investigation.

The present study had some limitations. First, as mentioned previously, the accuracy of our equations may be challenged. However, these equations were developed for one group of subjects and further validated in another validation group. Therefore, we believe our equations are reliable under the condition of a relatively large sample size, which could decrease equation biases. Second, this is a cross-sectional study. Compared to a longitudinally designed study, our results are less persuasive. Further studies designed to follow subjects along a time course are required to validate our findings. Finally, it should be noted that our findings were derived from Chinese participants and two centers. Therefore, our findings should be carefully extrapolated to other ethnic groups.

## 5. Conclusions

In conclusion, we found that age was negatively related to IR and GE within both sexes of Chinese individuals with normal FPG levels (90 mg/dL) and BMIs (men: 23 kg/m^2^, women: 22 kg/m^2^). However, only the FPIS was negatively related to age in women. GE had the tightest relationship, followed by the FPIS and IR. Therefore, the importance of GE should be re-evaluated. These results should be interpreted with caution because of the small sample size.

## Figures and Tables

**Table 1:** Demographic characteristics, biochemical data, and diabetes factors (DFs) of the study subjects.

	Men	Women	*p*
n	43	126	
Age (years)	33.1 ± 18.8	36.3 ± 19.5	0.078
Body mass index (BMI) (kg/m^2^)	23 ± 0.3	22 ± 0.3	0.271
Systolic blood pressure (SBP) (mmHg)	116 ± 14	113 ± 17	0.055
Diastolic blood pressure (DBP) (mmHg)	71 ± 9	71 ± 11	0.993
Fasting plasma glucose (FPG) (mg/dL)	90.0 ± 0.000 ^a^	90.0 ± 0.000 ^a^	
High-density lipoprotein cholesterol (HDL-C) (mmol/dL)	1.130 ± 0.306	1.348 ± 0.357	<0.001
Low-density lipoprotein cholesterol (LDL-C) (mmol/dL)	3.166 ± 0.757	3.162 ± 0.783	0.971
Triglyceride (TG) (mmol/dL)	1.217 ± 0.683	1.081 ± 0.608	0.083
Hemoglobin (10^3^/μL)	15.0 ± 1.0	13.0 ± 1.0	<0.001
White blood cell count (10^3^/μL)	6.285 ± 1.745	6.023 ± 1.463	0.179
Platelet count (10^3^/μL)	222 ± 49	247 ± 54	<0.001
Glucose effectiveness (GE) (10^−2^ ∙dL ∙min^−1^ ∙kg^−1^)	0.019 ± 0.002	0.019 ± 0.002	0.204
Insulin resistance (IR) (10^−4^ ∙min^−1^ ∙pmol^−1^ ∙L^−1^)	3.684 ± 0.014	3.685 ± 0.013	0.809
Log transformation of first-phase insulin secretion (Log_FPIS) (μU/min)	2.067 ± 0.159	1.950 ± 0.186	<0.001
Log transformation of second-phase insulin secretion (Log_SPIS) (pmol/mmol)	−1.222 ± 0.020	−1.224 ± 0.021	0.271

Data are shown as the mean ± standard deviation (SD). *p* < 0.05 indicates there are significant differences between men and women. ^a^ means base.

**Table 2:** Pearson correlation between diabetes factors in male and female subjects.

	r	*p*
Male		
Log transformation of the first-phase insulin secretion	−0.008	0.929
Log transformation of the second-phase insulin secretion	−0.028	0.762
Insulin resistance	−0.391	<0.001
Glucose effectiveness	−0.667	<0.001
Female		
Log transformation of the first-phase insulin secretion	−0.238	0.003
Log transformation of the second-phase insulin secretion	0.096	0.240
Insulin resistance	−0.240	0.003
Glucose effectiveness	−0.780	<0.001

## Data Availability

Data can be provided upon request to the author by e-mail.
